# Supplementary material for: Plasmodium vivax transcriptomes reveal stage-specific chloroquine response and differential regulation of male and female gametocytes
Source: Nat Commun. 2019 Jan 22;10:371. doi: 10.1038/s41467-019-08312-z (PMC6342968; doi:10.1038/s41467-019-08312-z)

**Supplementary Information**

*Plasmodium vivax* transcriptomes reveal stage-specific chloroquine response and differential regulation of male and female gametocytes

Kim *et al.*

**Supplementary Figure 1: Correlation between parasitemia and proportion of RNA-seq reads mapped to the *P. vivax* genome**. Each dot represents one infection and is displayed according to the proportion of reads mapped to the *P. vivax* genome (x-axis, in %) and the parasitemia determined by microscopy (y-axis, in parasite/µL) (Pearson's R=0.37, p=0.06).

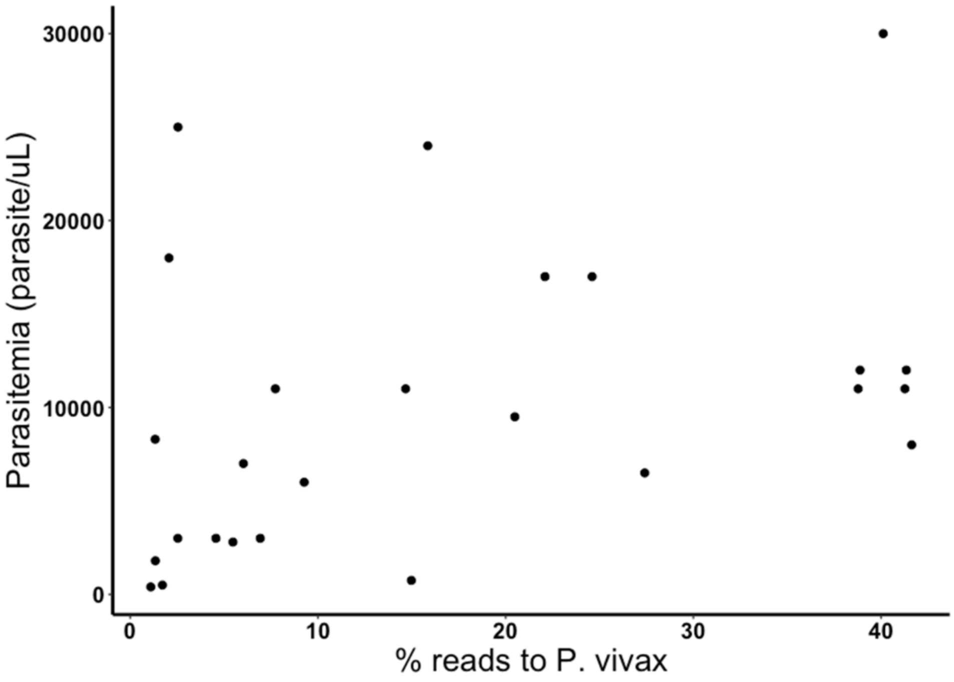

**Supplementary Figure 2: Principal component analysis of the 26 clinical infections based on the parasite gene expression profiles.** Each dot represents one infection (before treatment) and is displayed according to the value of the principal components 1 (x-axis) and 2 (y-axis).

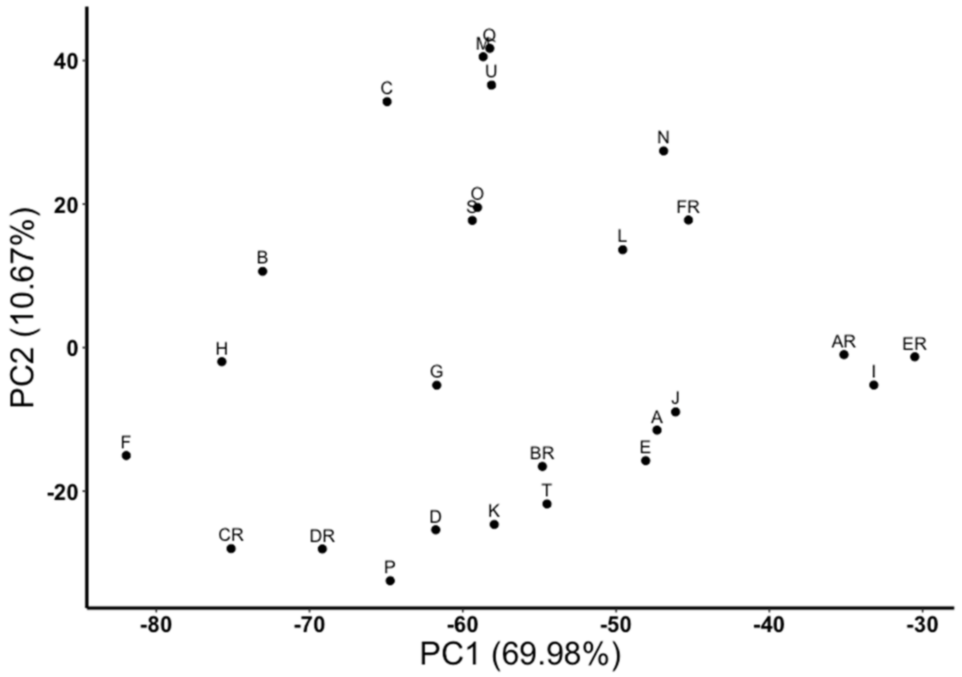

**Supplementary Figure 3: Validation of the gene expression deconvolution approach.** The figure shows the results of gene expression deconvolution analyses of (**A**) synchronized *P. falciparum* cultures (in hours, RNA-seq data from Otto et al., 2010) and (**B**) mock mixtures of *P. berghei* stages (%schizonts, scRNA-data from Reid et al., 2018). See Methods for details.

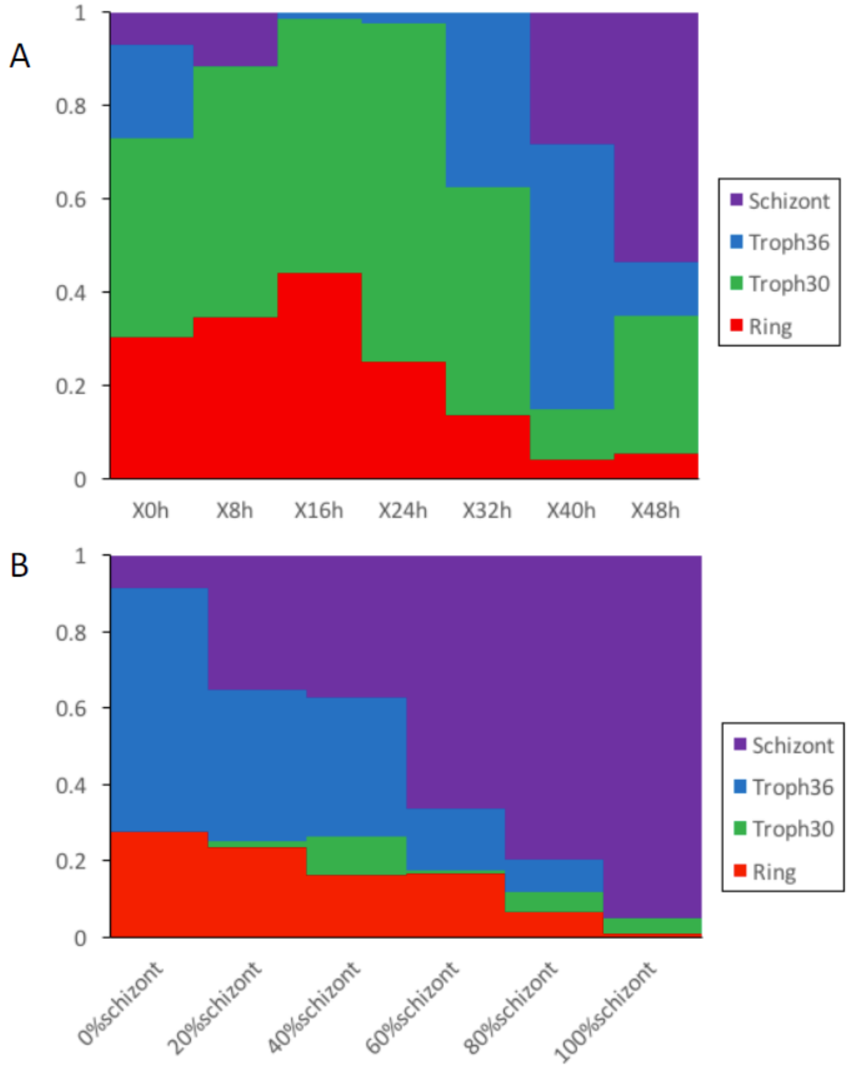

**Supplementary Figure 4: Effect of chloroquine treatment.** The scatterplot shows, for each infection (black dot), the proportion of remaining parasites (in %) eight hours after chloroquine administration as determined by microscopy (x-axis) and using the proportion of RNA-seq reads mapped to *P. vivax* (y-axis).

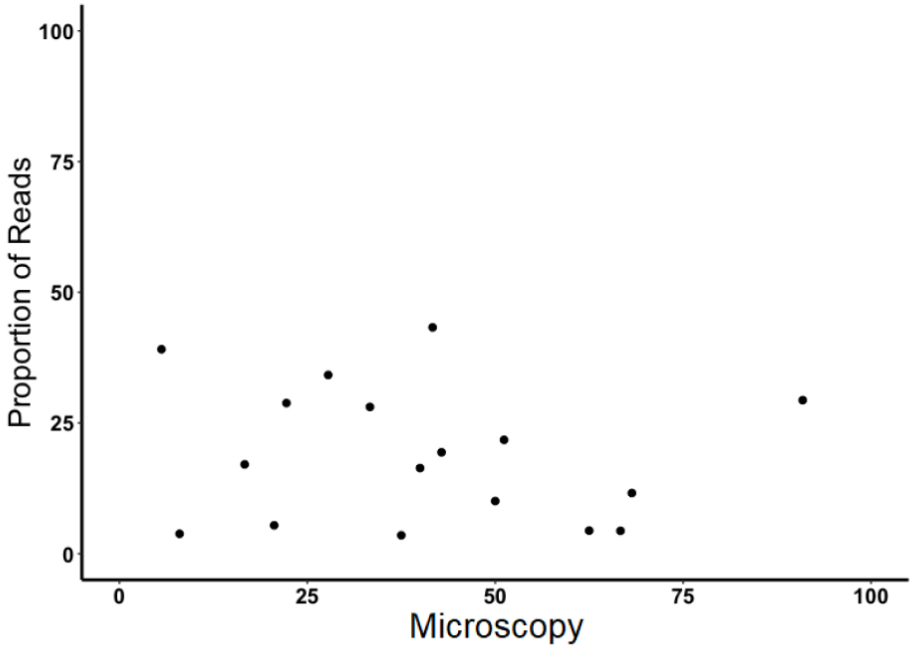

**Supplementary Figure 5: Stage composition eight hours after chloroquine administration.** The figure shows the results of the gene expression deconvolution analysis of patient blood samples eight hours after chloroquine treatment. (The gene expression deconvolution for the S infection was not successful and is therefore not shown).

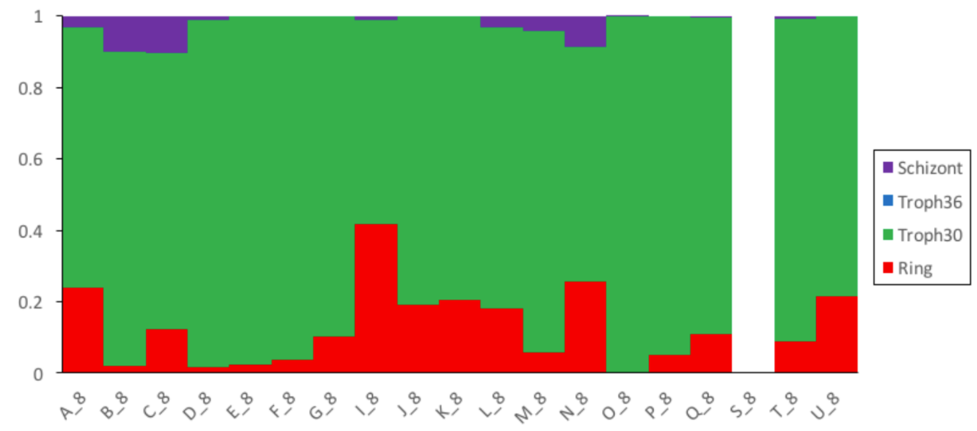

**Supplementary Figure 6: Effect of chloroquine on parasite gene expression.** Principal component analysis of the parasite gene expression profiles before (round dots) and eight hours after chloroquine administration (arrow heads). Each arrow represents the change in overall parasite gene expression of one infection.

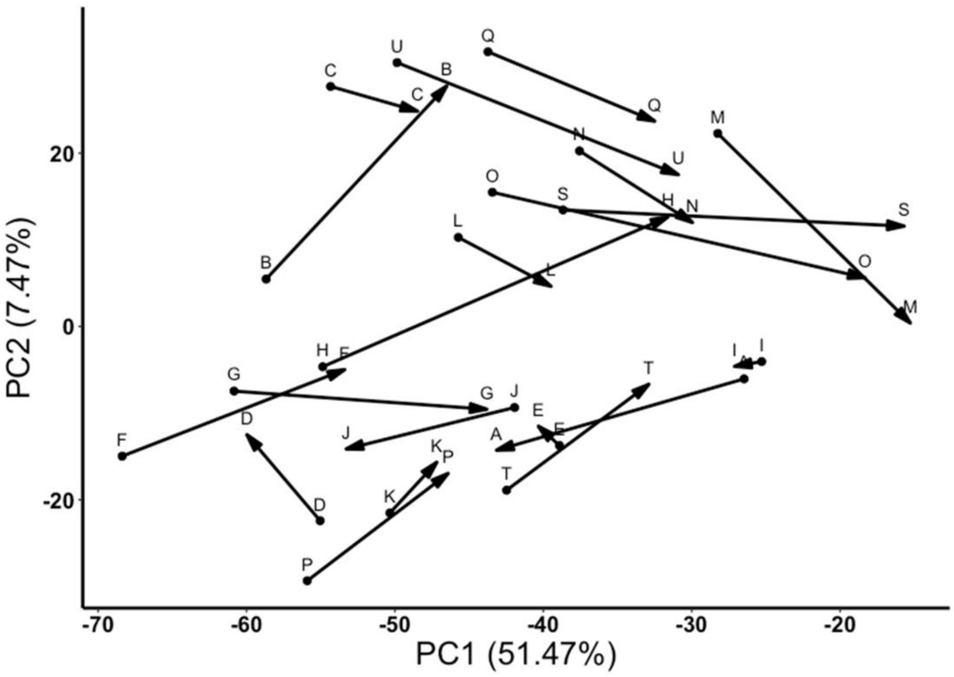

**Supplementary Figure 7: Proportion of spliced isoforms of drug resistance candidate genes.** The figures show, for each infection (displayed along the x-axis), the proportion of isoforms spliced (y-axis, in %) for (**A**) PvCRT and (**B**) PvMDR1 for each primary infection.

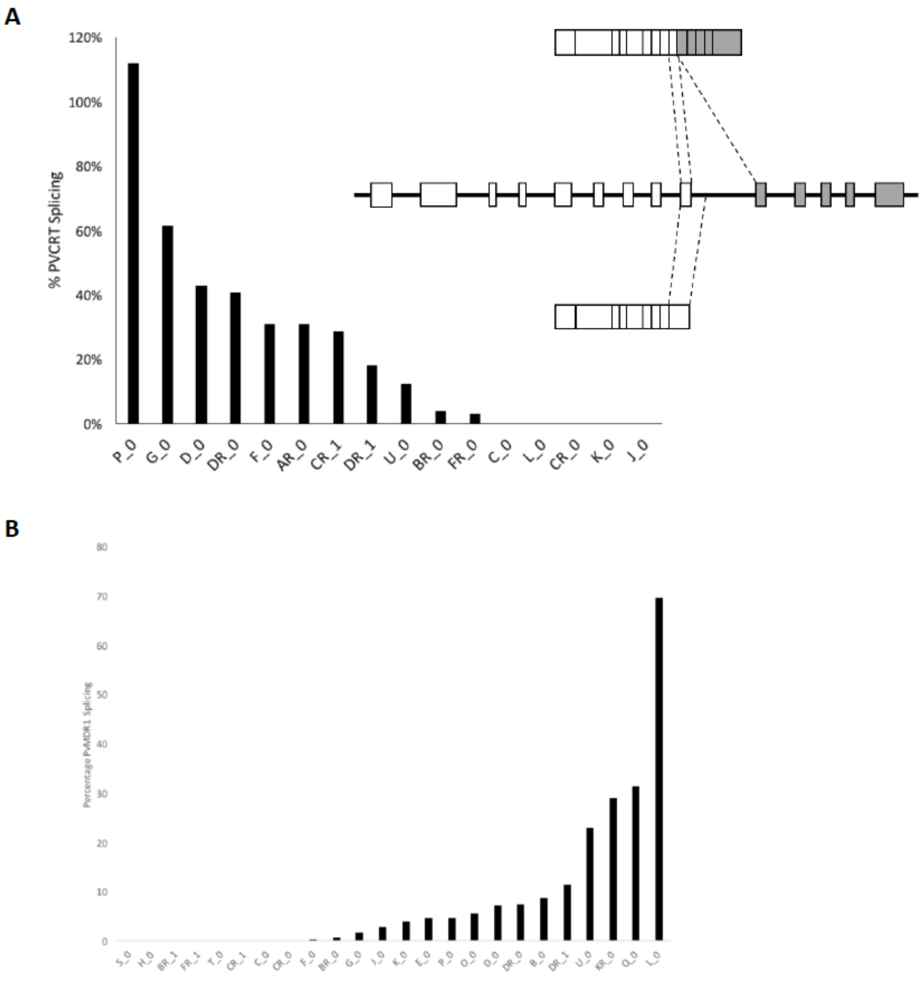

Supplement: Supplementary file 1 — Supplementary Information [file 41467_2019_8312_MOESM1_ESM.pdf]
