# Supplementary material for: Plasmodium vivax transcriptomes reveal stage-specific chloroquine response and differential regulation of male and female gametocytes
Source: Nat Commun. 2019 Jan 22;10:371. doi: 10.1038/s41467-019-08312-z (PMC6342968; doi:10.1038/s41467-019-08312-z)
Supplement: Supplementary file 3 — Description of Additional Supplementary Files [file 41467_2019_8312_MOESM3_ESM.pdf]

Description of Additional Supplementary Files

File Name: Supplementary Data 1
Description: Sequencing and alignment statistics of each infection

File Name: Supplementary Data 2
Description: List of P. vivax genes whose expression levels are correlated with the expression of known gametocyte genes.

File Name: Supplementary Data 3
Description: Genes differentially expressed after chloroquine treatment (FDR<0.1).

File Name: Supplementary Data 4
Description: Gene expression signatures of the different developmental stages obtained by gene expression deconvolution.
