# Supplementary material for: Plasmodium vivax transcriptomes reveal stage-specific chloroquine response and differential regulation of male and female gametocytes
Source: Nat Commun. 2019 Jan 22;10:371. doi: 10.1038/s41467-019-08312-z (PMC6342968; doi:10.1038/s41467-019-08312-z)
Supplement: Supplementary file 8 — Reporting Summary [file 41467_2019_8312_MOESM8_ESM.pdf]

nature research

Corresponding author(s): David Serre

Last updated by author(s): Dec 19, 2018

Reporting Summary

Nature Research wishes to improve the reproducibility of the work that we publish. This form provides structure for consistency and transparency in reporting. For further information on Nature Research policies, see Authors & Referees and the Editorial Policy Checklist.

Statistics

For all statistical analyses, confirm that the following items are present in the figure legend, table legend, main text, or Methods section.

n/a	Confirmed	
☐	☒	The exact sample size (n) for each experimental group/condition, given as a discrete number and unit of measurement
☒	☐	A statement on whether measurements were taken from distinct samples or whether the same sample was measured repeatedly
☐	☒	The statistical test(s) used AND whether they are one- or two-sided<br>*Only common tests should be described solely by name; describe more complex techniques in the Methods section.*
☐	☒	A description of all covariates tested
☒	☐	A description of any assumptions or corrections, such as tests of normality and adjustment for multiple comparisons
☐	☒	A full description of the statistical parameters including central tendency (e.g. means) or other basic estimates (e.g. regression coefficient) AND variation (e.g. standard deviation) or associated estimates of uncertainty (e.g. confidence intervals)
☒	☐	For null hypothesis testing, the test statistic (e.g. F, t, r) with confidence intervals, effect sizes, degrees of freedom and P value noted<br>*Give P values as exact values whenever suitable.*
☒	☐	For Bayesian analysis, information on the choice of priors and Markov chain Monte Carlo settings
☒	☐	For hierarchical and complex designs, identification of the appropriate level for tests and full reporting of outcomes
☒	☐	Estimates of effect sizes (e.g. Cohen's d, Pearson's r), indicating how they were calculated

Our web collection on statistics for biologists contains articles on many of the points above.

Software and code

Policy information about availability of computer code

Data collection	The codes and software used to collect and process data (including the version used and the parameters) are indicated in the Methods and Code availability sections
Data analysis	The codes and software used to analyses data (including the version used and the parameters) are indicated in the Methods and Code availability sections

For manuscripts utilizing custom algorithms or software that are central to the research but not yet described in published literature, software must be made available to editors/reviewers. We strongly encourage code deposition in a community repository (e.g. GitHub). See the Nature Research guidelines for submitting code & software for further information.

Data

Policy information about availability of data

All manuscripts must include a data availability statement. This statement should provide the following information, where applicable:

- Accession codes, unique identifiers, or web links for publicly available datasets
- A list of figures that have associated raw data
- A description of any restrictions on data availability

All data generated are freely available through SRA (accession number PRJNA378759)

Field-specific reporting

Please select the one below that is the best fit for your research. If you are not sure, read the appropriate sections before making your selection.

☒ Life sciences ☐ Behavioural & social sciences ☐ Ecological, evolutionary & environmental sciences

Life sciences study design

All studies must disclose on these points even when the disclosure is negative.

Sample size	We analyze blood samples from all the patients included in the study.
Data exclusions	No exclusion.
Replication	No replication due to the study design.
Randomization	No randomization as the study relies rely on i) analyses of the diversity among patients and ii) paired comparisons (before and after treatment)
Blinding	No blinding due to the study design

Reporting for specific materials, systems and methods

We require information from authors about some types of materials, experimental systems and methods used in many studies. Here, indicate whether each material, system or method listed is relevant to your study. If you are not sure if a list item applies to your research, read the appropriate section before selecting a response.

Materials & experimental systems

n/a	Involved in the study	
☒	☐	Antibodies
☒	☐	Eukaryotic cell lines
☒	☐	Palaeontology
☒	☐	Animals and other organisms
☐	☒	Human research participants
☒	☐	Clinical data

Methods

n/a	Involved in the study	
☒	☐	ChIP-seq
☒	☐	Flow cytometry
☒	☐	MRI-based neuroimaging

Human research participants

Policy information about studies involving human research participants

Population characteristics	Cambodian individuals with uncomplicated clinical vivax malaria were enrolled in this study. Our population characteristics reflect the burden of this disease in Cambodia (mostly males 20-35 yo).
Recruitment	Individuals presenting with fever (or history of fever within 48 hours) and positive for P. vivax by RDT and PCR were included in this study.
Ethics oversight	The study was approved by the Cambodian National Ethics Committee for Health Research (038 NECHR 24/02/14) and registered at ClinicalTrials.gov (NTC02118090).

Note that full information on the approval of the study protocol must also be provided in the manuscript.
